# Early Cancer Detection via Multi-microRNA Profiling of Urinary Exosomes Captured by Nanowires

**DOI:** 10.1021/acs.analchem.4c02488

**Published:** 2024-10-18

**Authors:** Takao Yasui, Atsushi Natsume, Takeshi Yanagida, Kazuki Nagashima, Takashi Washio, Yuki Ichikawa, Kunanon Chattrairat, Tsuyoshi Naganawa, Mikiko Iida, Yotaro Kitano, Kosuke Aoki, Mika Mizunuma, Taisuke Shimada, Kazuya Takayama, Takahiro Ochiya, Tomoji Kawai, Yoshinobu Baba

**Affiliations:** † Department of Life Science and Technology, Tokyo Institute of Technology, Nagatsuta 4259, Midori-ku, Yokohama 226-8501, Japan; ‡ Institute of Quantum Life Science, National Institutes for Quantum Science and Technology (QST), Anagawa 4-9-1, Inage-ku, Chiba 263-8555, Japan; § Institute of Nano-Life-Systems, Institutes of Innovation for Future Society, Nagoya University, Furo-cho, Chikusa-ku, Nagoya 464-8603, Japan; ∥ Craif Inc., 3-38-14-3 Hongo, Bunkyo-ku, Tokyo 113-0033, Japan; ⊥ Kawamura Medical Society, Gifu 501-3144, Japan; # Department of Applied Chemistry, Graduate School of Engineering, The University of Tokyo, 7-3-1 Hongo, Bunkyo-ku, Tokyo 113-8656, Japan; ¶ The Institute of Scientific and Industrial Research, Osaka University, 8-1 Mihogaoka, Ibaraki, Osaka 567-0047, Japan; □ Research Institute for Electronic Science (RIES), Hokkaido University, N21W10, Kita, Sapporo, Hokkaido 001-0021, Japan; ○ Department of Biomolecular Engineering, Graduate School of Engineering, Nagoya University, Furo-cho, Chikusa-ku, Nagoya 464-8603, Japan; △ Department of Neurosurgery, School of Medicine, Nagoya University, 65 Tsurumai-cho, Showa-ku, Nagoya 466-8550, Japan; ▲ Department of Molecular and Cellular Medicine, Tokyo Medical University, 6-7-1 Nishishinjyuku, Shinjuku-ku, Tokyo 160-0023, Japan

## Abstract

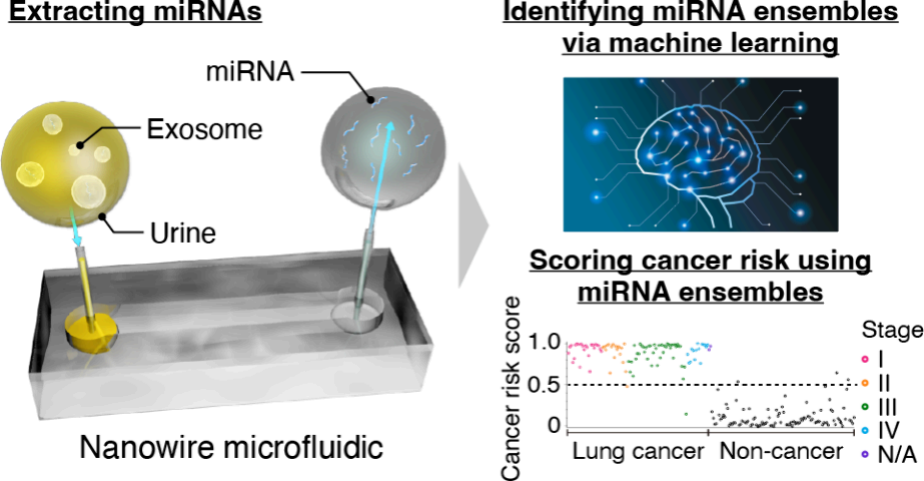

Multiple microRNAs encapsulated in extracellular vesicles (EVs) including exosomes, unique subtypes of EVs, differ in healthy and cancer groups of people, and they represent a warning sign for various cancer scenarios. Since all EVs in blood cannot be transferred from donor to recipient cells during a single blood circulation, kidney filtration could pass some untransferred EVs from blood to urine. Previously, we reported on the ability of zinc oxide nanowires to capture EVs based on surface charge and hydrogen bonding; these nanowires extracted massive numbers of microRNAs in urine, seeking cancer-related microRNAs through statistical analysis. Here, we report on the scalability of the nanowire performance capability to comprehensively capture EVs, including exosomes, in urine, extract microRNAs from the captured EVs *in situ*, and identify multiple microRNAs in the extracted microRNAs differing in noncancer and lung cancer subjects through machine learning-based analysis. The nanowire-based extraction allowed the presence of about 2500 species of urinary microRNAs to be confirmed, meaning that urine includes almost all human microRNA species. The machine learning-based analysis identified multiple microRNAs from the extracted microRNA species. The ensembles could classify cancer and noncancer subjects with the area under the receiver operating characteristic curve of 0.99, even though the former were staged early.

## Introduction

MicroRNA (miRNA) ensembles are formed by some of the miRNA species in blood, and they can represent a warning sign for various cancer scenarios since they have been found to differ in healthy and cancer groups of people.^1^ Circulating miRNAs in blood are mostly encapsulated in extracellular vesicles (EVs),^2,3^ which are well-known information carriers in body fluids.^4,5^ Cancer cells can regulate biological processes of other cells via the circulating miRNAs, such as promoting cancer cell dormancy,^6^ promoting tumorigenesis,^7^ and breaking down blood brain barriers.^8^ Considering the fact that cancer-related events include complex biological processes, cancer-related events should correlate with some sets of miRNA species that refer to miRNA ensembles. Recent studies have demonstrated that serum miRNA ensembles showed a promising feature that allows discriminating between healthy and cancer subject groups.^9−14^

**Figure 1:**
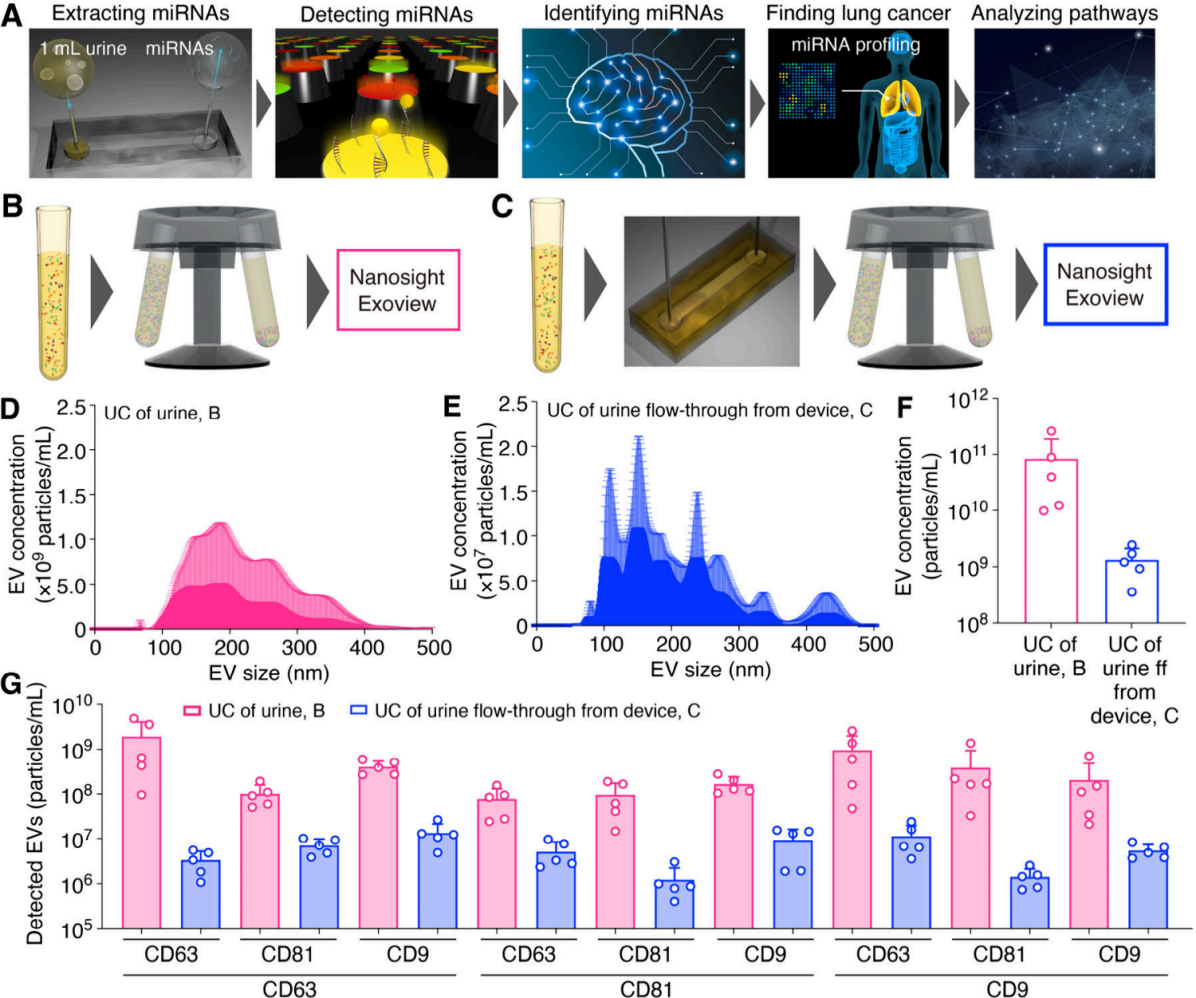
Nanowire-assisted extraction of exosomal miRNAs in urine. (A) Schematic illustrations for miRNA extraction from urine using a nanowire device, nanowire-extracted miRNA detection using microarrays with 2565 species probes, identification of microRNA ensembles based on fluorescence intensity analysis of each miRNA species using a logistic regression-modeled classifier, pathway analysis of identified miRNA ensembles, and lung cancer detection using identified miRNA ensembles. (B) Schematic illustrations for EV analysis in raw urine. (C) Schematic illustrations for EV analysis in flow-through urine from the device. (D) EV size distribution of urine samples after ultracentrifugation (UC). (E) EV size distribution of UC of urine flow-through from the device. (F) EV concentration of UC of urine and UC of urine flow-through from the device. (G) Membrane protein expression levels of UC of urine and UC of urine flow-through from the device. The line below the *x*-axis represents the captured antibody type, and the line above it indicates the corresponding fluorescent-labeled antibody. For instance, the CD81 line above the CD63 line signifies that EVs captured with the anti-CD63 antibody were detected using the fluorescently labeled anti-CD81 antibody. (D-G) Error bars show the standard deviation for a series of measurements, *N* = 5.

Urinary miRNA ensembles, which are blood byproducts, have the potential to serve as a cancer warning sign, the same as the blood miRNA ensembles do. In view of blood circulation time^15^ and EV diffusion time from one side of the blood vessels to the other, which are determined by the diffusion coefficient (∼15 μm^2^ s^–1^),^16^ blood viscosity,^17^ and vessel diameter,^18^ not all miRNAs in blood are transferred from donor to recipient cells in a single blood circulation, and therefore, it is implied that some miRNAs are nonselectively filtered out by the kidneys. Noninvasiveness of urine collection is superior to that of blood; however, finding urinary miRNA ensembles with cancer-related regulations has been limited due to the small numbers of identified miRNA species in urine (∼300).^19^ Previously, we exceeded the conventional number of identified miRNA species in urine: using nanowires we captured urinary EVs comprehensively,^20−23^ and found about 1300 miRNA species in a 1 mL sample of single-donor urine.^24^ Most species of urinary miRNAs are simply undetectable by the ultracentrifugation method due to their low abundance.^25^ Given this, some well-known miRNAs, such as miR-21,^26,27^ have not been assigned as potential cancer-related urinary miRNAs. This is thought to be due to the fact that the discovered number of urinary miRNA species has been around half of the current number of reported miRNA species in humans, ∼2600 species,^26^ and there are individual differences in the types of miRNA species extracted. Two key issues remain for identifying urinary miRNA ensembles with cancer-related regulations: (i) extracting urinary miRNAs comprehensively; and (ii) constructing miRNA ensembles warning of cancer. Here, we propose a method to address the issues for identifying urinary miRNA ensembles through a combination of nanowire-based miRNA extraction and machine learning analysis (Figure 1A).

We previously utilized a nanowire device to extract a wide range of urinary miRNAs that could identify specific types of carcinomas.^24^ Among oxide nanowires (SiO_2_, TiO_2_, and ZnO), ZnO nanowires, which have the strongest positively charged surface, have shown an efficient capability to capture EVs from urine based on electrostatic interaction and hydrogen bonding.^21,28,29^ Furthermore, the concentration of captured EV using nanowires correlates with CD63 expression level, and we found that urinary EVs could serve as a biomarker for the presence of brain tumors.^30^ These findings indicate that urinary EVs have the potential to be a promising tool for detecting specific carcinomas. Therefore, urinary miRNAs mostly encapsulated in EVs can also be biomarkers for specific types of cancer. In this study, we presented conclusive evidence supporting the utilization of urinary miRNAs derived from urinary EVs, including exosomes, unique subtypes of EVs sized 40–200 nm and characterized by CD63.^31^ And, we used the identified urinary miRNA ensembles to distinguish lung cancer and noncancer subjects. These urinary miRNAs show great potential as promising tools for early cancer detection.

## Experimental Section

### Fabrication Procedure for Nanowires

Details of the nanowire fabrication can be found elsewhere.^19^ For fabrication of zinc oxide (ZnO) nanowires, a 140 nm thick Cr layer was deposited on a Si substrate with the channel pattern using a sputtering system (Elionix Inc.). The Cr layer was thermally oxidized at 400 °C for 2 h and served as a seed layer for ZnO nanowire growth. The ZnO nanowires were grown by immersing the substrate in a solution mixture of 15 mM hexamethylenetetramine (HMTA, Wako Pure Chemical Industries, Ltd.) and 15 mM zinc nitrate hexahydrate (Thermo Fisher Scientific Inc.) at 95 °C for 3 h. Poly(dimethylsiloxane) (PDMS) (Silpot 184, Dow Corning Corp.) was poured onto the nanowire-grown substrate, followed by curing and peeling off the PDMS from the substrate (nanowire-transferred PDMS). Then, the nanowire growth was carried out by immersing the nanowire-transferred PDMS in a mixed solution of 15 mM HMTA and 15 mM zinc nitrate hexahydrate at 95 °C for 3 h (nanowire-embedded PDMS). Next, the nanowire-embedded PDMS substrate was bonded to a herringbone-structured PDMS substrate having a microchannel (2 mm width, 2 cm length, and 50 μm height) with a 12-μm high herringbone structure. Finally, the herringbone-structured substrate was connected to poly(ether ether ketone) (PEEK) tubes (O.D. 0.5 mm × I.D. 0.26 mm, 10 cm length; Institute of Microchemical Technology Co., Ltd.) for an inlet and an outlet (Figure S1).

### MicroRNA Extraction from Urine Using Nanowires

ZnO nanowires could extract urinary miRNAs, comprehensively, in flow-through fractions in the following two steps: first, the positively charged surface of the ZnO nanowires could capture negatively charged EVs and free-floating miRNAs (ff-miRNAs) in urine via electrostatic interactions and hydrogen bonding; and second, lysis buffer could release EV-encapsulated miRNAs from captured EVs and ff-miRNAs from the surface of the nanowires. After centrifuging commercially available urine biospecimens (ProteoGenex, Inc., Biomedica CRO, and East West Biopharma LLC.) and subject urine specimens obtained before surgery (15 min, 4 °C, 3000 *g*) to remove apoptotic bodies, each 1 mL urine sample aliquot was introduced into the nanowire array at a flow rate of 50 μL/min using a syringe pump (KDS-200, KD Scientific Inc.). Then, 1 mL of lysis buffer (Cell Lysis Buffer M, Wako Pure Chemical Industries, Ltd.; 20 mM Tris-HCl (pH 7.4), 200 mM sodium chloride, 2.5 mM magnesium chloride, 0.05 w/v% NP-40 substitute) was introduced using the syringe pump (flow rate, 50 μL/min) into the nanowire array to extract miRNAs.

### Microarray Analysis of miRNA Expression

The SeraMir Exosome RNA Purification Column Kit (System Biosciences, Inc.) was used according to the manufacturer’s instruction manual to purify the miRNA solution extracted by lysis buffer. 15 μL of purified miRNA was analyzed for miRNA profiling using a microarray and the 3D-Gene Human miRNA Oligo Chip ver.21 (Toray Industries), which was designed to detect 2565 miRNAs sequences registered in miRBase release 21 (http://www.mirbase.org/) based on fluorescent signals. The background noise was subtracted from the fluorescence signal intensity, and this intensity was then calibrated using the global normalization method with the median value as 25. The globally normalized intensities for each sample were log 2 transformed.

### Identifying Urinary miRNA Ensembles

The classifiers were based on a logistic regression-modeled classifier as follows:
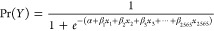
1where *Y* is a predicted objective variable, *x* is fluorescence intensity of each miRNA species, β is weight coefficient of each miRNA species, and α is the intercept. In this model, β and α were estimated from each fluorescence intensity of the nanowire-extracted urinary miRNA species by using machine learning. We defined a value of *Y* below 0.5 as a noncancer subject, while *Y* more than or equal to 0.5 was a cancer subject. The classifier solved an optimization problem for the least-squares error term and the L1 regularization term simultaneously when fitting the logistic regression classifier; λ acted as an adjuster between the two terms.

The classifier could explore the dominant factors that determine miRNAs related to cancer. By adjustment of λ during the classifier derivation, it was possible to increase or decrease the number of explanatory variables that made up the logistic regression classifier. More specifically, increasing λ increased the number of explanatory variables with an absolute value of β equal to 0, which decreased the number of explanatory variables and the number of miRNA species in the classifier. On the other hand, decreasing λ increased the number of explanatory variables with an absolute value of β more than 0, which increased the number of explanatory variables and the number of miRNA species in the classifier. The weight coefficient, β, acted as a contribution factor for each miRNA species. By narrowing down the number of explanatory variables in this way, it was possible to get a clue to narrow down the number of dominant factors that had a decisive impact on cancer. At the same time, it was also possible to get a clue to identify factors that had no decisive impact on cancer and were only incidentally expressed in the available data. We used λ = 1 unless otherwise stated, which showed high area under the curve, sensitivity, and specificity.

After setting the value of λ, the classifier output the explanatory variable weights (weights for each miRNA species) β and intercept α, which did not have an absolute value of 0 through L1 regularization. When cross-validation was performed, the attribute selection results for the number of learning iterations performed were output, and the average of β and α obtained for the number of learning iterations performed were taken, and only those whose absolute value of the average was greater than the threshold of 0.01 were the final attribute selection results. We used the 20-fold cross-validation unless otherwise stated, which employed a large fraction of the data sets for training to maintain statistically high accuracy. This output file covered both the primary attribute selection results for each round of cross-validation and the final attribute selection results. The aforementioned functionalities were integrated into a single application by AISoftware Inc.

A receiver operating characteristic (ROC) curve was depicted by plotting sensitivity (true positive rate) on the *y*-axis vs specificity (false positive rate) on the *x*-axis for the test set values in the training set using BellCurve for Excel (Social Survey Research Information Co., Ltd.). The area under the ROC curve (AUROC) was used to discriminate the presence or absence of cancer subjects among all subjects. An AUROC of 0.5 represented a test with no discriminating ability, while an AUROC of 1.0 represented a test with a complete discrimination ability.

### Pathway Analysis

In total, 338 KEGG pathways (Release 96.0, October 1, 2020), consisting of 8430 unique genes (background set), were used in this analysis. A contingency table was generated by counting the number of genes that overlap the background set and the list of miRNA targets of interest for every KEGG pathway. The pathway enrichment analysis of miRNA targets was done using Fisher’s exact tests. The *p* values were adjusted for multiple comparisons using the Benjamini Hochberge procedure. The FDR threshold of 0.05 was used to identify the significantly enriched pathways.

## Results and Discussion

### Nanowire-Based Extraction of miRNAs in Urine

To validate the EV capture by a nanowire device, we extracted EVs for urine samples by comparing the standard method, ultracentrifugation (UC), and our nanowire device (Figures 1B and 1C). The UC of urine and UC of urine flow-through from the device were characterized by using Nanosight and Exoview. The size distributions of EVs for both methods were quite similar, ranging from 100 to 500 nm, suggesting that the EVs were not ruptured after flowing through the nanowire device (Figures 1D and 1E). When we investigated the concentration of EVs in each method, the UC of urine showed about a 60-fold higher concentration than the UC of urine flow-through from the device, suggesting that the nanowire device captured a large number of EVs (Figure 1F). And, if we hypnotized that the UC of urine separated all of the EVs in urine, the capture percentage of the nanowire device (*C*_0_ – *C*_ff_)/*C*_0_ was around 99% where *C*_0_ is EV concentration for UC of urine and *C*_ff_ is EV concentration for UC of urine flow-through from the device. Furthermore, as expected, the membrane proteins (CD63, CD81, and CD9) for UC of urine were more highly expressed than those of urine flow-through from the device, confirming that the EVs were captured inside the nanowire device (Figure 1G). And, we supposed that the nanowire device could capture all EV subtypes, including exosomes, due to the decrease of all membrane protein expression levels in UC of urine flow-through from the device. These results confirmed that we could extract a large number of EVs from urine using the nanowire device and that the extracted subjects were suitable for further downstream analysis.

### Comprehensive Extraction of Urinary miRNAs

**Figure 2:**
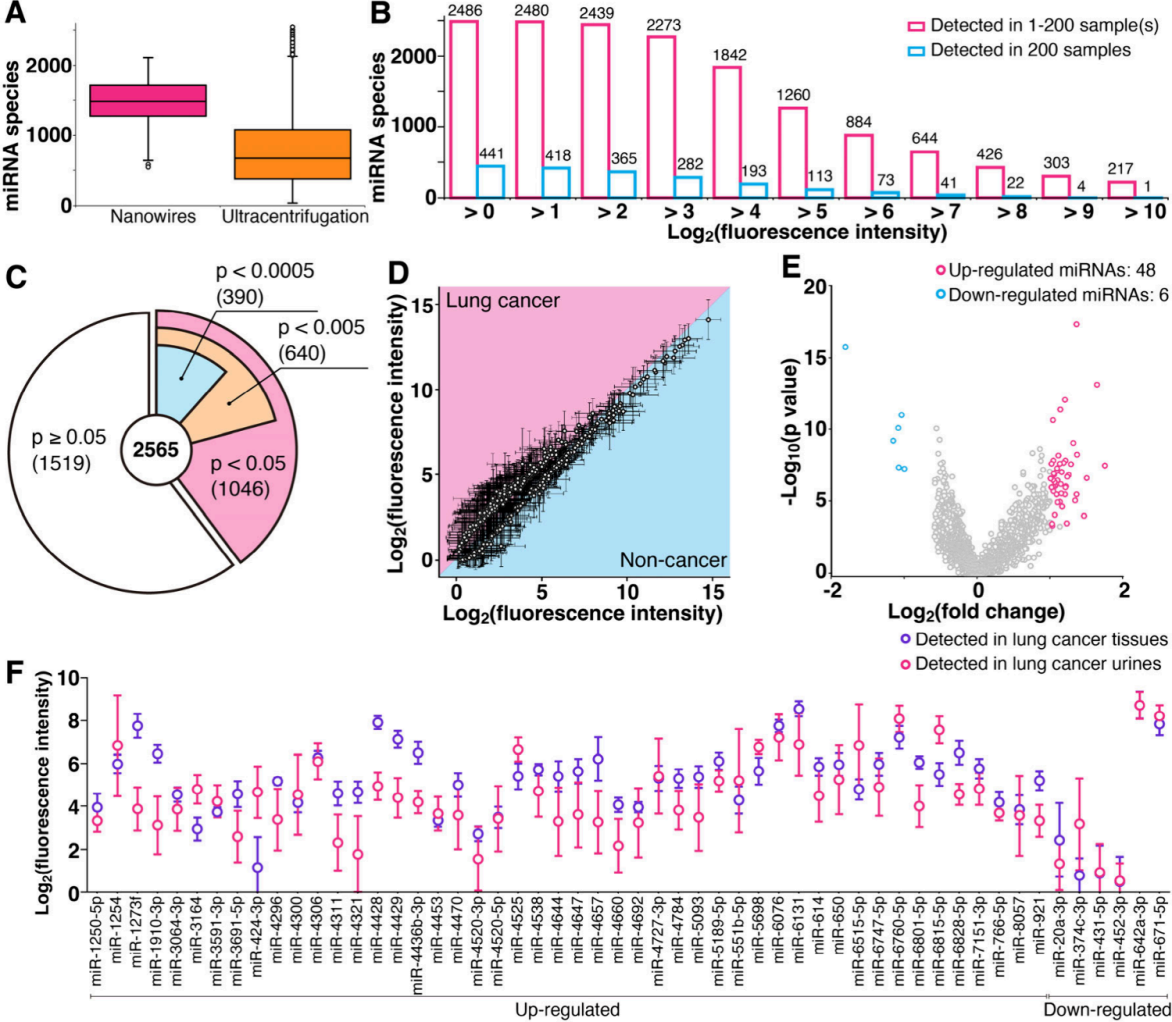
Comprehensive extraction of urinary miRNAs. (A) Box plot of extracted miRNA species using nanowire-based extraction from urine (pink, this work) and UC-based extraction from serum (orange, data from reference^9^). Colored box lengths represent the interquartile range (first to third quartiles); the line in the center of each box represents the median value, and the bars show the data range (maximum to minimum). Error bars show the standard deviation for a series of measurements, *N* = 200 and *N* = 4046 for nanowire and UC methods, respectively. (B) Histogram of nanowire-extracted urinary miRNA species found in at least one sample (pink) and in all samples (cyan). (C) Pie chart of nanowire-extracted urinary miRNA species and p values showing statistically significant differences between lung cancer and noncancer subjects. (D) Scatter plot of fluorescence intensity of miRNAs with *p* values of less than 0.0005 extracted from lung cancer urine samples vs noncancer urine samples. Each point corresponds to a different miRNA species. The boundary between pink and cyan represents the same level of miRNA expression for the two samples. Error bars show the standard deviation for a series of measurements (*N* = 100). (E) Volcano plot highlighting significant miRNA species. Each point corresponds to a different miRNA species. The *x*- and *y*-axes represent the logarithm of the fluorescence ratio between lung cancer and noncancer subjects and the logarithm of p values showing statistically significant differences between lung cancer and noncancer subjects, respectively. The featured miRNAs have −log_10_(*p* value) of more than 3.30 and log_2_(fold change) of more than 1.00 or less than −1.00. (F) Comparison between fluorescence intensities of featured miRNAs (54 species) in lung cancer subjects extracted from samples of tissues and urine. Error bars show the standard deviation for a series of measurements (*N* = 8).

We confirmed whether nanowire-based miRNA extraction could demonstrate comprehensive extraction of urinary miRNAs by increasing the number of urine samples (Figure 2). When the number of species of extracted serum miRNAs in 4046 samples was confirmed by UC,^9^ the median number of species of extracted serum miRNAs was about 700 (Figure 2A), but 2565 miRNA species were confirmed in serum, suggesting that the strategy of increasing the number of urine samples would allow us to confirm the presence of as many as 2565 miRNA species in urine. We used 200 urine samples, 100 from patients who had been diagnosed with stages I to IV lung cancer and 100 from noncancer subjects (Table S1). A screening for nanowire-based miRNA extraction from these 200 samples revealed that the median number of extracted urine miRNAs was around 1500 species (Figure 2A). The screening also revealed the presence of 2486 miRNA species in the urine samples; 441 species were commonly found in all samples, and the remaining 2045 miRNAs varied among individuals (Figure 2B). These results suggest that the previously found number of miRNA species (∼1300) could not assign well-known miRNAs as potentially cancer-related miRNAs due to individual differences. Compared to the report on miRNA species in serum extracted by UC^9^ that 30 species were commonly found in 4046 subjects (and 120 species were commonly found in a randomly selected 200 from among the 4046 subjects), we saw that nanowire-based miRNA extraction finds a larger number of miRNA species in urine than UC-based extraction does in serum. From the analysis of 200 urine samples, we concluded that urine has almost all human miRNA species and these miRNAs are nonselectively filtered out by the kidneys.

The fact that urine has almost all human miRNA species led us to consider the following possible scenario: urinary miRNA species might be transferred from cancer tissues via blood circulation. To support the scenario, we confirmed there were statistically significant differences between lung cancer and noncancer subjects. Results of a nonparametric test, the Mann–Whitney U test, showed that between lung cancer and noncancer subject groups, the differences in fluorescence intensities of 1046, 640, and 390 species were *p* < 0.05, 0.005, and 0.0005, respectively (Figure 2C). Among the 390 miRNAs with *p* < 0.0005, 193 urinary miRNAs were abundant in the urine samples of cancer patients and 197 urinary miRNAs were abundant in the urine samples of noncancer subjects (Figure 2D). Constructing the volcano plot for these miRNAs, we obtained 54 urinary miRNAs that were statistically significant: 48 were up-regulated and 6 were down-regulated miRNAs in lung cancer subject urine (Figure 2E). All of the statistically significant miRNAs were also found in cancer tissues in the same donor samples (Figure 2F). These results highlighted that urinary miRNA species come from cancer tissues via blood circulation.

### Identifying Urinary miRNA Ensembles

**Figure 3:**
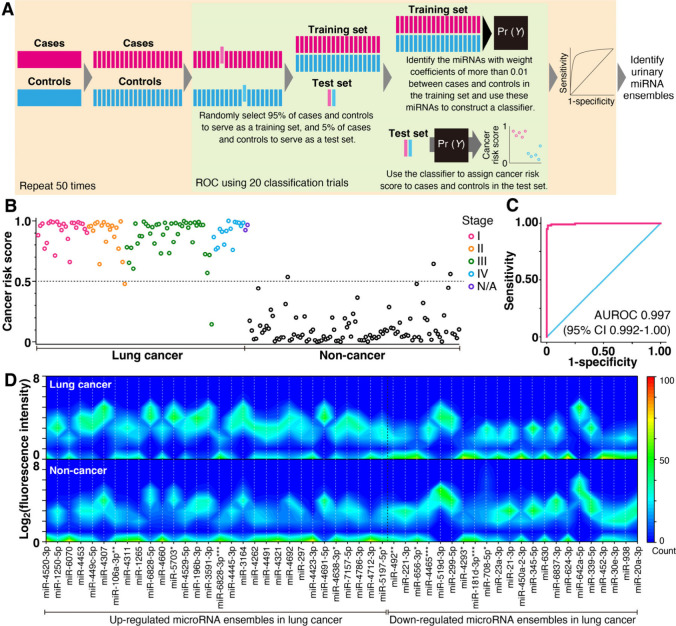
Identifying urinary miRNA ensembles. (A) Summary of analytical approaches used to classify and identify samples based on urinary miRNA ensembles. (B) Cancer risk scores and (C) AUROC curve for 100 lung cancer and 100 noncancer subjects obtained using one urinary miRNA ensemble. Since the classifier model is based on a logistic regression, the threshold for lung cancer risk is 0.5; among noncancer subjects the value is below 0.5; and for cancer subjects, it is more than or equal to 0.5. N/A represents stage as unknown. (D) Heat maps of logarithmic fluorescence intensity vs the one urinary miRNA ensemble used in the classification of Figures 3B and 3C for lung cancer (upper) and noncancer (lower) subjects. Red and blue indicate 100 and 0 overlapped data, respectively.

Next, we identified urinary miRNA ensembles from comprehensively extracted miRNAs for classification into lung cancer and noncancer subject groups (Figure 3), since some of the extracted miRNAs included cancer tissue-originating miRNAs. Although we found statistically significant miRNA species, their individual contributions to the classification were unequal. Then, to determine the most suitable urinary miRNA ensembles, we gave each miRNA species a contribution factor, that is, a weight coefficient, and we constructed a logistic regression classifier using supervised machine learning with cross-validation based on fluorescence intensities of nanowire-extracted urinary miRNAs (Figure 3A). We identified one suitable urinary miRNA ensemble composed of 53 miRNA species (light green area in Figure 3A, Table S2). Although there is no specific urinary EV miRNA database, we validated the identified miRNA species using existing EV miRNA databases ExoCarta and Vesiclepedia. We found up to 70% of the 53 miRNA species in these databases, confirming that the identified miRNAs come from EVs. Using a classifier based on this urinary miRNA ensemble, we obtained cancer risk scores (Figure 3B) and a receiver operating characteristic (ROC) curve^32^ for 100 lung cancer and 100 noncancer subjects which could statistically distinguish the cancer patients (area under the receiver operating characteristic curve, AUROC, 0.997; 95% confidence interval, 95% CI, 0.992–1.00) (Figure 3C). Repeating the cross-validation 50 times (light orange area in Figure 3A) identified a urinary miRNA ensemble composed of 52 miRNA species with classification performance of accuracy of 97.3%, sensitivity of 98.2%, and specificity of 96.5%. Heat maps of fluorescence intensities for the miRNA ensemble between lung cancer and noncancer subjects showed different expression patterns (Figure 3D and Figure S2). The combination of comprehensively extracted urinary miRNAs and machine learning analysis allows miRNA ensembles in urine to be extracted for cancer detection.

### Pathway Analysis and Early Staged Cancer Detection

**Figure 4:**
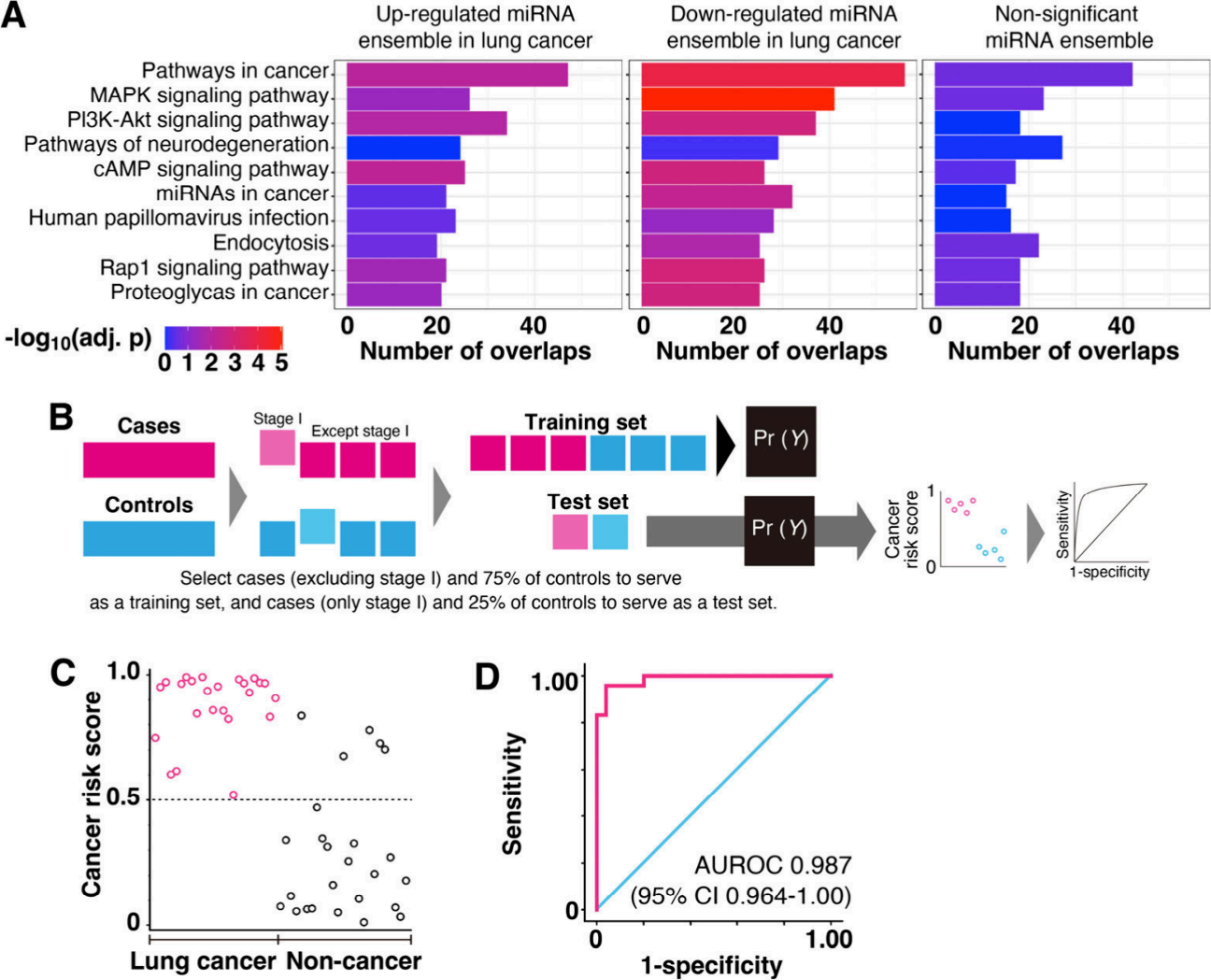
Pathway analysis and early staged cancer detection using identified miRNA ensembles. (A) Top 10 pathways with the largest number of total overlaps with miRNA targets from three groups (up- and down-regulated, nonsignificant) are shown in a bar plot, with the *x*-axis showing the extent of overlaps for each list and colors showing the significance in enrichment analysis. The p values were adjusted for multiple comparisons by using the Benjamini Hochberge procedure. (B) Summary of analytical approaches used to classify and identify stage I samples based on urinary miRNA ensembles. (C) Cancer risk scores and (D) AUROC curve for 24 stage I lung cancer and 25 noncancer subjects obtained using one urinary miRNA ensemble. This ensemble was identified using miRNA expression data from lung cancer subjects, excluding those of stage I, and 75 noncancer subjects.

Since urinary miRNAs were transferred from cancer tissues and some of the miRNAs made an ensemble for cancer classification, we conducted pathway analyses for each miRNA species of the identified ensembles (Figure 4A). Including a nonsignificant miRNA ensemble selected by the classifier (Figure S3, Table S3), we highlighted the top 10 pathways with the largest number of total overlaps with miRNA targets from three groups (up- and down-regulated miRNA ensembles and the nonsignificant miRNA ensemble). Among the 10 pathways, most of them were relevant to carcinogenesis. Compared to the nonsignificant miRNA ensemble (Figure S4), the up- and down-regulated miRNA ensembles in lung cancer subjects had a higher relationship to carcinogenesis. Moreover, the down-regulated microRNA ensemble in lung cancer subjects showed a larger significant difference of cancer-related pathways than the up-regulated miRNA ensemble. The pathway analysis highlighted the finding that the urinary miRNA ensembles were composed of miRNA species that significantly correlated to carcinogenesis.

Finally, to confirm whether urinary miRNA ensembles are associated with carcinogenesis independently of staging, we identified another urinary miRNA ensemble, which was supervised by miRNA fluorescence intensities from 76 cancer subjects, excluding those in stage I, and 75 noncancer subjects (Figure 4B). This urinary miRNA ensemble was composed of 30 miRNA species (Table S4). Using a classifier based on this urinary miRNA ensemble, we got cancer risk scores (Figure 4C) and the AUROC curve for 24 stage I lung cancer and 25 noncancer subjects which could statistically distinguish the early staged cancer patients (AUROC, 0.987; 95% CI, 0.964–1.00) (Figure 4D). Even though we supervised the classifier using cancer subjects (excluding stage I) and noncancer subjects, the classifier could distinguish between lung cancer of stage I and noncancer subjects. This result implies that the identified urinary miRNA ensemble is related to carcinogenesis. Since circulating tumor DNA predicts early staged lung cancer risk at around 40–60%,^33^ urinary miRNA ensembles would have sufficient potential for liquid biopsy use in early stage lung cancer prediction.

### Nanowire-Based Extraction and Machine Learning-Based Analyses of miRNAs in Urine

The EV size distribution of the UC of urine flow-through from the device (Figure 1E) was similar to that of the UC of urine (Figure 1D). The membrane protein expression levels (CD63, CD81, and CD9) of the UC of urine flow-through from the device were lower than those of the UC of urine (Figure 1F). These results suggested that the nanowire device nonselectively captured intact EVs, including exosomes, leading to a large number of extracted miRNA species (2486 species). This number represented around 96% of miRNAs from serum, implying that miRNAs are transferred via blood circulation and nonselectively filtered out by the kidneys. Moreover, after constructing the volcano plot, we found statistically significant miRNAs differing between cancer and noncancer subjects. Therefore, we concluded that almost all miRNAs existed in urine and had the potential to identify cancer-related functions similar to those in serum.

We were able to obtain a large number of miRNA species using the nanowire device. This allowed us to identify the urinary miRNA ensemble that was used to calculate the cancer risk scores. Using this ensemble, we could classify subjects into lung cancer and noncancer groups with an AUROC of 0.997. In addition to identifying a urinary miRNA ensemble for the classification of lung cancer and noncancer subjects, we conducted pathway analyses for each miRNA species of the identified ensembles, leading to obtaining the classifier to distinguish between lung cancer of stage I and noncancer subjects. Thus, we believe that urinary miRNA ensembles have the potential for liquid biopsy in early stage cancer detection.

### Identifying Urinary miRNA Ensembles for Classifying Brain Tumor, Lung Cancer, and Noncancer Subjects

We have identified urinary miRNA ensembles via the combination of nanowire-based miRNA extraction and machine learning-based analysis, and using these ensembles, we achieved early stage cancer classification with high accuracy, sensitivity, and specificity. Our nanowire-based miRNA extraction for 200 urine samples found around 2500 miRNA species in urine that had not been found using conventional methods. The machine learning-based analysis helped us to identify miRNA ensembles with highly accurate classification performance from the comprehensively extracted urinary miRNAs. The identified miRNA ensembles could distinguish lung cancer and noncancer subjects with more than 95% accuracy, sensitivity, and specificity. Furthermore, the urinary miRNA ensemble for distinguishing three classifications among brain tumor, lung cancer and noncancer subjects (Table S5) with 86% sensitivity and 93% specificity indicated a brain tumor patient with cerebral hemorrhage after surgery as a noncancer subject (Figure S5). Since we could classify brain tumors, which are anatomically farthest from the urinary system, from urine samples of just 1 mL, we can reasonably expect applicability of this concept to 10 types of cancer (Figure S6). This requires further study since some of the classifications showed low accuracy due to the small number of subjects (there were only 30 samples for each cancer type). Although we need to undertake further performance trials for this concept, the present results are encouraging us to develop urine-based liquid biopsies for future medical applications and to develop urinary miRNA-based diagnoses for timely medical checkups of the presence of cancer presence.

## Conclusion

In this study, we present the utilization of urinary miRNAs derived from urinary EVs, including exosomes, captured by nanowires. The nanowires could capture more than 99% of the EVs in urine, and the captured EVs had expression of the membrane proteins (CD63, CD81, and CD9). Moreover, the nanowire-based method showed the ability to extract about 2500 species of urinary miRNAs. Compared with serum miRNA species, the urinary miRNA species extracted by the nanowire-based method showed almost the same number of miRNA species, meaning that urine includes almost all human miRNAs. And, we used the identified urinary miRNA ensembles to distinguish lung cancer and noncancer subjects with an AUROC of 0.997; even when the lung cancer was stage I, an AUROC of 0.987 was achieved. These results suggested that miRNAs are transferred via blood circulation and nonselectively filtered out by kidneys. Furthermore, we used identified miRNA ensembles to distinguish three classifications among brain tumor, lung cancer, and noncancer subjects with 86% sensitivity and 93% specificity. Although a higher number of samples is required, these urinary miRNAs show great potential as promising tools for early cancer detection.

## Supporting Information Available

Photo of the experimental setup (Figures S1); Histograms of extracted miRNA species (Figure S2); The miRNA species with an absolute value over 0.01 of the weight coefficients vs number of subjects or logarithmic fluorescence intensity (Figure S3); Identifying nonsignificant miRNA ensembles (Figure S4); Identifying urinary miRNA ensembles for classification of brain tumor, lung cancer, and noncancer subjects (Figure S5); Applicability of miRNA ensemble concept to 10 types of cancer (Figure S6); Participant characteristics for lung cancer and noncancer subjects (Tables S1); A urinary miRNA ensemble composed of 53 miRNA species for classification between 100 lung cancer and 100 noncancer subjects (Table S2); A nonsignificant urinary miRNA ensemble composed of 59 miRNA species for classification between 100 lung cancer and 100 noncancer subjects (Table S3); A urinary miRNA ensemble composed of 40 miRNA species for classification between 24 stage I lung cancer and 25 noncancer subjects (Table S4); Participant characteristics for brain tumor, lung cancer, and noncancer subjects (Table S5). (PDF)
