# Supplementary material for: Early Cancer Detection via Multi-microRNA Profiling of Urinary Exosomes Captured by Nanowires
Source: Anal Chem. 2024 Oct 18;96(43):17145–53. doi: 10.1021/acs.analchem.4c02488 (PMC11525924; doi:10.1021/acs.analchem.4c02488)
Supplement: Supplementary file 1 — ac4c02488_si_001.pdf [file ac4c02488_si_001.pdf]

Supporting Information

Early cancer detection via multi-microRNA profiling of urinary exosomes captured by nanowires

Takao Yasui[1-4], Atsushi Natsume[3-5]*, Takeshi Yanagida[6,7]*, Kazuki Nagashima[8], Takashi Washio[7], Yuki Ichikawa[4], Kunanon Chattrairat[1], Tsuyoshi Naganawa[9], Mikiko Iida[3], Yotaro Kitano[10], Kosuke Aoki[10], Mika Mizunuma[4], Taisuke Shimada[2], Kazuya Takayama[4], Takahiro Ochiya[11], Tomoji Kawai[7], and Yoshinobu Baba[2,3]**

[1]Department of Life Science and Technology, Tokyo Institute of Technology, Nagatsuta 4259, Midori-ku, Yokohama 226-8501, Japan.

[2]Institute of Quantum Life Science, National Institutes for Quantum Science and Technology (QST), Anagawa 4-9-1, Inage-ku, Chiba 263-8555, Japan.

[3]Institute of Nano-Life-Systems, Institutes of Innovation for Future Society, Nagoya University, Furo-cho, Chikusa-ku, Nagoya 464-8603, Japan.

[4]Craif Inc., 3-38-14-3 Hongo, Bunkyo-ku, Tokyo 113-0033, Japan.

[5]Kawamura Medical Society, Gifu 501-3144, Japan

[6]Department of Applied Chemistry, Graduate School of Engineering, The University of Tokyo, 7-3-1 Hongo, Bunkyo-ku, Tokyo 113-8656, Japan.

[7]The Institute of Scientific and Industrial Research, Osaka University, 8-1 Mihogaoka, Ibaraki, Osaka 567-0047, Japan.

[8]Research Institute for Electronic Science (RIES), Hokkaido University, N21W10, Kita, Sapporo, Hokkaido 001-0021, Japan.

[9]Department of Biomolecular Engineering, Graduate School of Engineering, Nagoya University, Furo-cho, Chikusa-ku, Nagoya 464-8603, Japan.

[10]Department of Neurosurgery, School of Medicine, Nagoya University, 65 Tsurumai-cho, Showa-ku, Nagoya 466-8550, Japan.

[11]Department of Molecular and Cellular Medicine, Tokyo Medical University, 6-7-1 Nishishinjyuku, Shinjuku-ku, Tokyo 160-0023, Japan.

*Corresponding authors: yasuit@bio.titech.ac.jp; atsushi.natsume@mirai.nagoya-u.ac.jp; yanagida@g.ecc.u-tokyo.ac.jp; babaymtt@chembio.nagoya-u.ac.jp.

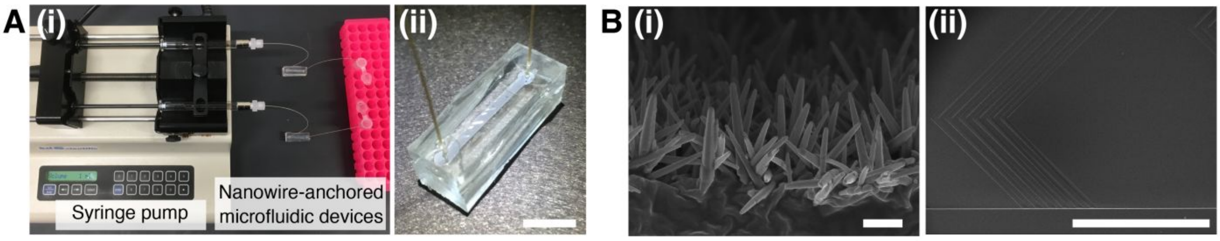

Figure S1. (A) (i) Photo of the experimental setup, including a syringe pump, two nanowire-anchored microfluidic devices, and two 1.5 mL tubes. (ii) Photo of the nanowire-anchored microfluidic device; scale bar, 1 cm. The device consisted of nanowires, which were anchored into polydimethylsiloxane (PDMS), and the PDMS microfluidic herringbone structure. (B) SEM image of nanowires anchored into PDMS; scale bar, 1 μm. (ii) SEM image of the PDMS microfluidic herringbone structure; scale bar, 1 mm.

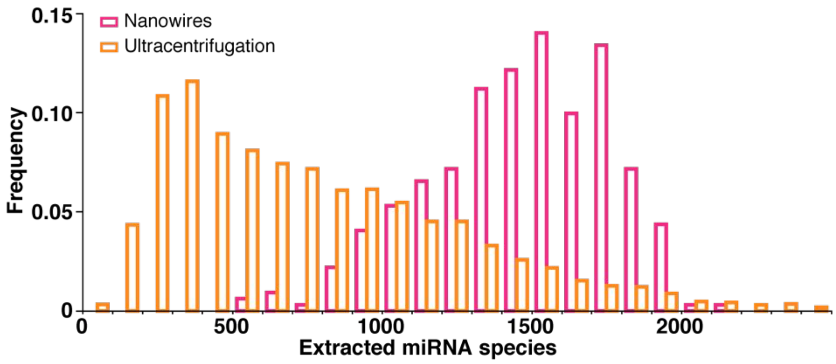

Figure S2. Histograms of extracted miRNA species obtained when using nanowire-based extraction (pink) and ultracentrifugation-based extraction (orange).

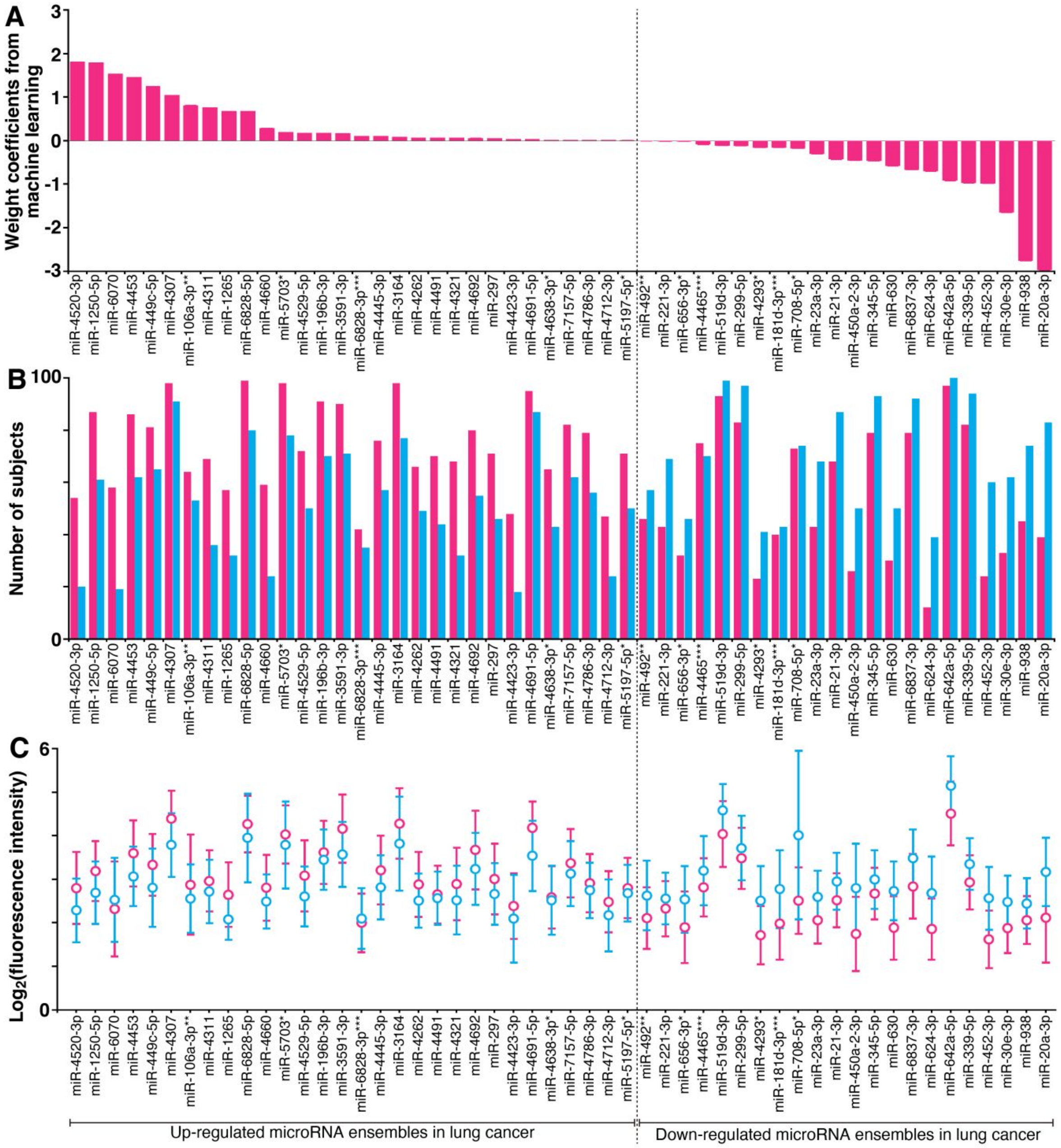

Figure S3. (A) The miRNA species with an absolute value over 0.01 of the weight coefficients vs. (B) number of subjects or (C) logarithmic fluorescence intensity. The asterisks indicate p values for each miRNA: none, $p < 0.0005$; one asterisk, $p < 0.005$; two asterisks, $p < 0.05$; and three asterisks, $p \geq 0.05$. The black dotted line indicates a value of more than (left) or less than (right) 0 for the weight coefficient. (A) Error bars show the standard deviation for a series of analyses (N=3). (B) Each miRNA species was not found in all urine samples examined, *e.g.*, only 54 lung cancer subjects had miR-4520-3p and 20 non-cancer subjects had miR-4520-3p. (c) The data points of fluorescence intensities for each miRNA species were calculated only from the number of subjects for each miRNA, *e.g.*, N=54 for miR-4520-3p of lung cancer subjects and N=20 for miR-4520-3p of non-cancer subjects. (B, C) Pink and cyan colors represent lung cancer and non-cancer subjects.

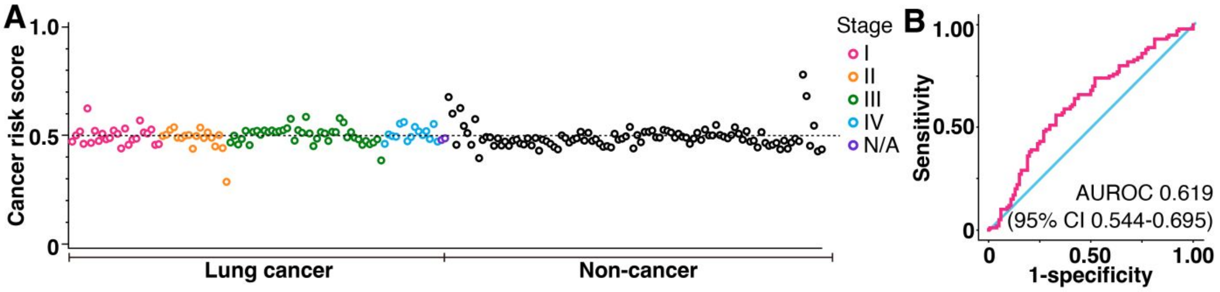

Figure S4. Identifying non-significant miRNA ensembles. (A, B) Cancer risk scores and AUROC curve for 100 lung cancer and 100 non-cancer subjects obtained using the non-significant urinary miRNA ensemble. The threshold for lung cancer risk is 0.5; among non-cancer subjects the value is below 0.5; and for cancer subjects it is more than or equal to 0.5. N/A represents stage as unknown. Repeating the cross validation 50 times (light orange area in Fig. 3a) provided classification performance of accuracy of 58.3%, sensitivity of 50.7%, and specificity of 65.9%.

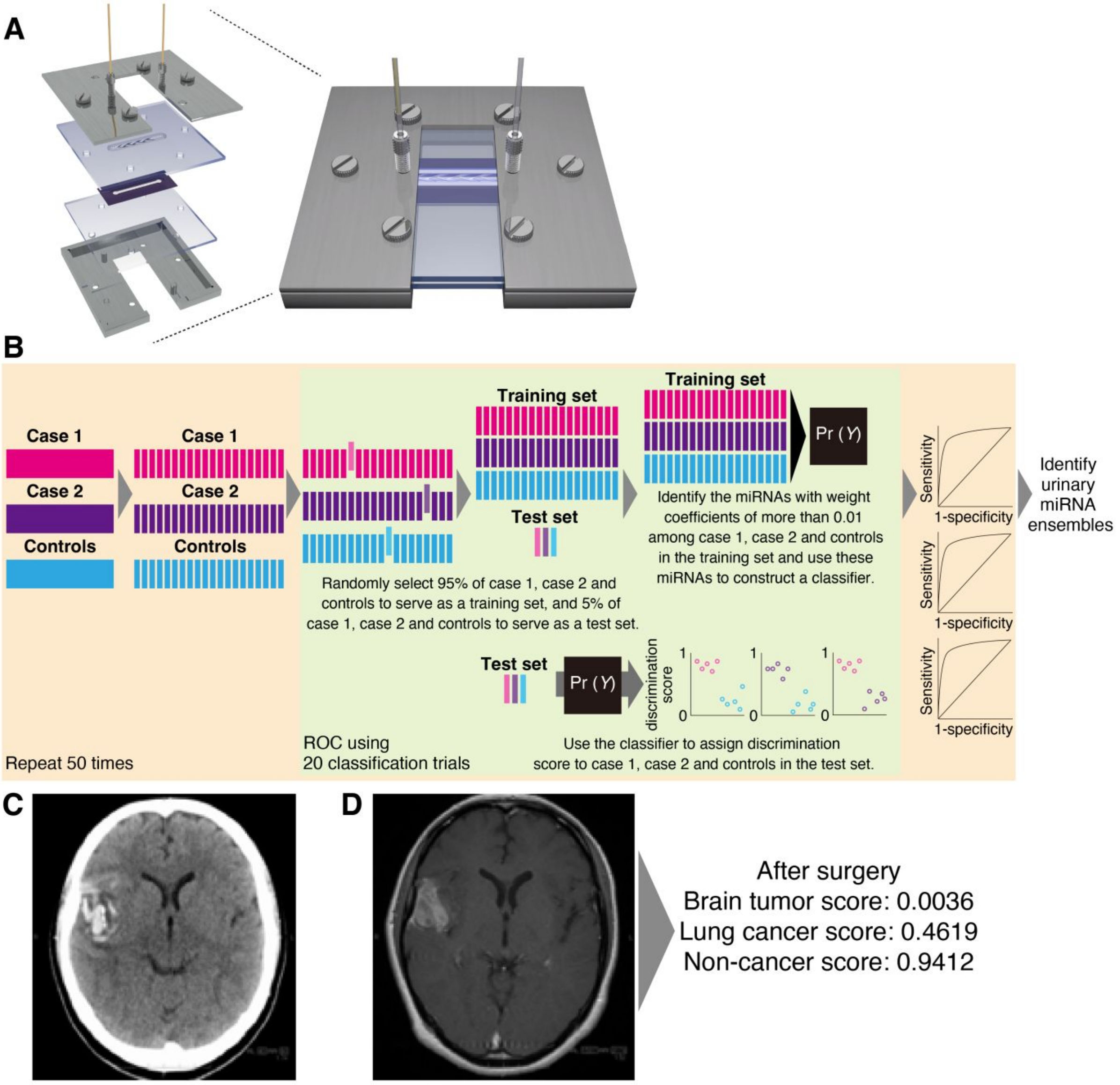

Figure S5. Identifying urinary miRNA ensembles for classification of brain tumor, lung cancer, and non-cancer subjects. (A) Schematic illustration of the nanowire microfluidic device used for classification of brain tumor, lung cancer, and non-cancer subjects. The device consisted of COP microfluidic resin, Si substrate with ZnO nanowires, COP resin, and a SUS holder. (B) Schematic illustration showing the identification of microRNA ensembles based on fluorescence intensity analysis of each miRNA species using a logistic regression-modeled 3-class classifier. (C) CT image of a 35-year-old female without a medical history complaining of headaches. Intracerebral hemorrhage at the right temporal lobe and subarachnoid hemorrhage in the right sylvian fissure with gyral calcification appeared in the image. (D) Post-contrast MRI for this female patient revealed a well circumscribed mass lesion at the right temporal lobe, suggesting presence of a brain tumor; the resected tumor was glioblastoma. After surgery, each discrimination score suggested she was a non-cancer subject.

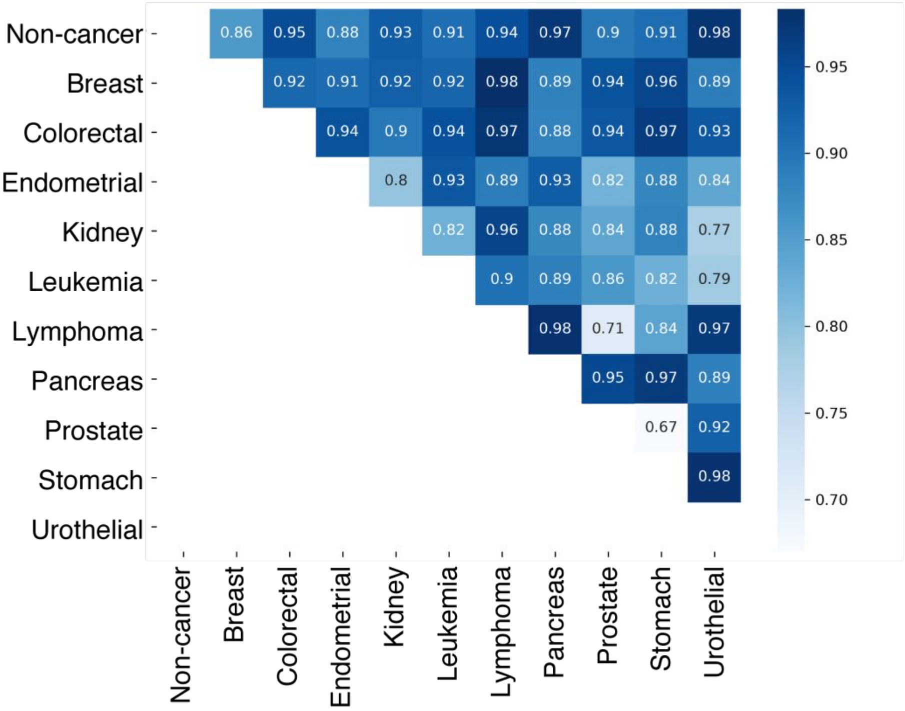

Figure S6. Applicability of miRNA ensemble concept to 10 types of cancer. Results of binary classifications among 10 cancer types and non-cancer subjects. Urine samples from 30 patients from each group were measured using the nanowire microfluidic device and classified with the binary logistic regression-model with leave-one-out cross-validation. The matrix shows the area under the ROC curve (AUROC) of the classification of two cohorts corresponding to the x and y-axes.

Table S1. Participant characteristics for lung cancer and non-cancer subjects.

Characteristics	Total (N=200)
Lung cancer, N	100
Median age, years (range)	61 (31-80)
Sex, N	
Male	94
Female	6
Clinical stage, N	
IA	10
IB	14
IIA	10
IIB	8
IIIA	30
IIIB	11
IV	15
Unknown	2
Smoking status, N	
Never smoked	6
Former smoker	47
Current smoker	47
Non-cancer controls, N	100
Median age, years (range)	41 (25-70)
Sex, N	
Male	58
Female	42
Smoking status, N	
Never smoked	100
Former smoker	0
Current smoker	0

Table S2. A urinary miRNA ensemble composed of 53 miRNA species for classification between 100 lung cancer and 100 non-cancer subjects.

MicroRNAs	Weight coefficient	p value	EV miRNAs confirmed by ExoCarta (released in July 2015) or Vesiclepedia (released in September 2023)
hsa-mir-4520-3p	1.818	1.14×10^{-7}	Confirmed
hsa-mir-1250-5p	1.788	2.49×10^{-8}	Confirmed
hsa-mir-6070	1.564	1.84×10^{-7}	Not confirmed
hsa-mir-4453	1.441	2.15×10^{-8}	Confirmed
hsa-mir-449c-5p	1.251	1.24×10^{-5}	Confirmed
hsa-mir-4307	1.043	2.26×10^{-9}	Not confirmed
hsa-mir-106a-3p	0.827	3.20×10^{-2}	Confirmed
hsa-mir-4311	0.740	8.68×10^{-7}	Confirmed
hsa-mir-1265	0.679	8.25×10^{-6}	Not confirmed
hsa-mir-6828-5p	0.673	8.76×10^{-5}	Not confirmed
hsa-mir-4660	0.282	1.35×10^{-7}	Confirmed
hsa-mir-4529-5p	0.187	1.72×10^{-5}	Not confirmed
hsa-mir-5703	0.180	9.17×10^{-4}	Confirmed
hsa-mir-3591-3p	0.177	1.42×10^{-8}	Confirmed
hsa-mir-196b-3p	0.146	2.33×10^{-4}	Confirmed
hsa-mir-6828-3p	0.104	4.11×10^{-1}	Not confirmed
hsa-mir-4445-3p	0.102	5.21×10^{-5}	Not confirmed
hsa-mir-4321	0.096	9.56×10^{-8}	Not confirmed
hsa-mir-3164	0.095	1.42×10^{-7}	Confirmed
hsa-mir-4262	0.083	3.86×10^{-4}	Not confirmed
hsa-mir-4491	0.078	2.54×10^{-4}	Not confirmed
hsa-mir-4692	0.072	2.76×10^{-6}	Confirmed
hsa-mir-4691-5p	0.069	6.25×10^{-9}	Confirmed
hsa-mir-297	0.049	3.12×10^{-5}	Confirmed
hsa-mir-4423-3p	0.044	1.71×10^{-6}	Not confirmed
hsa-mir-7157-5p	0.024	2.42×10^{-4}	Not confirmed
hsa-mir-4712-3p	0.020	3.25×10^{-4}	Confirmed

hsa-mir-4638-3p	0.019	3.05×10^{-3}	Confirmed
hsa-mir-2277-3p	0.013	8.54×10^{-4}	Confirmed
hsa-mir-5197-5p	0.012	2.70×10^{-3}	Not confirmed
hsa-mir-1322	0.011	1.47×10^{-4}	Confirmed
hsa-mir-492	-0.011	6.10×10^{-3}	Confirmed
hsa-mir-98-3p	-0.014	6.00×10^{-6}	Confirmed
hsa-mir-221-3p	-0.015	3.50×10^{-5}	Confirmed
hsa-mir-4465	-0.083	1.84×10^{-1}	Not confirmed
hsa-mir-519d-3p	-0.108	4.540×10^{-8}	Confirmed
hsa-mir-299-5p	-0.116	1.97×10^{-4}	Confirmed
hsa-mir-4293	-0.164	6.90×10^{-4}	Not confirmed
hsa-mir-708-5p	-0.168	8.35×10^{-4}	Confirmed
hsa-mir-181d-3p	-0.186	9.89×10^{-2}	Confirmed
hsa-mir-23a-3p	-0.308	8.69×10^{-7}	Confirmed
hsa-mir-21-3p	-0.440	2.68×10^{-7}	Confirmed
hsa-mir-450a-2-3p	-0.461	1.16×10^{-5}	Confirmed
hsa-mir-345-5p	-0.495	1.87×10^{-5}	Confirmed
hsa-mir-630	-0.585	6.05×10^{-5}	Confirmed
hsa-mir-6837-3p	-0.667	3.40×10^{-9}	Confirmed
hsa-mir-624-3p	-0.725	1.69×10^{-6}	Confirmed
hsa-mir-642a-5p	-0.926	5.73×10^{-10}	Confirmed
hsa-mir-452-3p	-0.960	6.05×10^{-10}	Confirmed
hsa-mir-339-5p	-0.967	1.96×10^{-7}	Confirmed
hsa-mir-30e-3p	-1.636	1.22×10^{-7}	Confirmed
hsa-mir-938	-2.770	1.60×10^{-7}	Not confirmed
hsa-mir-20a-3p	-2.977	1.95×10^{-16}	Confirmed

Table S3. A non-significant urinary miRNA ensemble composed of 59 miRNA species for classification between 100 lung cancer and 100 non-cancer subjects.

MicroRNAs	Weight coefficient	p value	EV miRNAs confirmed by ExoCarta (released in July 2015) or Vesiclepedia (released in September 2023)
hsa-miR-1228-5p	-	4.96×10^{-2}	Confirmed
hsa-miR-1237-5p	-	4.93×10^{-2}	Not confirmed
hsa-miR-1268a	-	9.50×10^{-1}	Confirmed
hsa-miR-1275	-0.925	2.31×10^{-6}	Confirmed
hsa-miR-1343-5p	-	1.71×10^{-1}	Confirmed
hsa-miR-1469	-	1.57×10^{-2}	Confirmed
hsa-miR-1908-5p	-	3.84×10^{-1}	Confirmed
hsa-miR-1914-3p	-	1.756×10^{-3}	Confirmed
hsa-miR-2861	-	1.68×10^{-5}	Confirmed
hsa-miR-3131	0.007	8.79×10^{-2}	Confirmed
hsa-miR-3178	-	9.41×10^{-1}	Confirmed
hsa-miR-3196	-	2.45×10^{-1}	Confirmed
hsa-miR-3197	-	1.23×10^{-1}	Confirmed
hsa-miR-3648	-	4.28×10^{-1}	Confirmed
hsa-miR-3656	-	5.55×10^{-1}	Confirmed
hsa-miR-3663-3p	0.001	7.41×10^{-1}	Confirmed
hsa-miR-3665	-	2.36×10^{-3}	Confirmed
hsa-miR-371a-5p	-1.186	2.97×10^{-6}	Confirmed
hsa-miR-3940-5p	-	1.47×10^{-1}	Confirmed
hsa-miR-4270	-	7.63×10^{-2}	Not confirmed
hsa-miR-4442	-	6.86×10^{-4}	Confirmed
hsa-miR-4463	-	4.50×10^{-2}	Confirmed
hsa-miR-4488	-	2.85×10^{-1}	Confirmed
hsa-miR-4492	-0.429	5.43×10^{-4}	Confirmed
hsa-miR-4508	-	2.51×10^{-2}	Confirmed
hsa-miR-4516	-	1.33×10^{-1}	Confirmed
hsa-miR-4534	-	3.84×10^{-2}	Confirmed

hsa-miR-4632-5p	-	2.70×10^{-1}	Confirmed
hsa-miR-4649-5p	0.010	7.02×10^{-1}	Not confirmed
hsa-miR-4675	-	4.63×10^{-2}	Not confirmed
hsa-miR-4689	-	4.07×10^{-5}	Confirmed
hsa-miR-4723-5p	-	9.83×10^{-2}	Not confirmed
hsa-miR-4728-5p	-	2.20×10^{-1}	Confirmed
hsa-miR-4741	0.002	4.01×10^{-1}	Confirmed
hsa-miR-4787-5p	-	1.03×10^{-3}	Confirmed
hsa-miR-5787	-0.002	1.01×10^{-4}	Confirmed
hsa-miR-6089	-	1.86×10^{-2}	Confirmed
hsa-miR-6090	-	3.10×10^{-1}	Confirmed
hsa-miR-6165	-	1.83×10^{-2}	Confirmed
hsa-miR-642b-3p	-0.025	4.29×10^{-5}	Confirmed
hsa-miR-6727-5p	-	2.90×10^{-2}	Confirmed
hsa-miR-6756-5p	0.009	6.02×10^{-1}	Not confirmed
hsa-miR-6757-5p	-	6.99×10^{-5}	Not confirmed
hsa-miR-6765-5p	-	4.54×10^{-3}	Confirmed
hsa-miR-6771-5p	-	9.30×10^{-2}	Not confirmed
hsa-miR-6785-5p	-	3.41×10^{-1}	Confirmed
hsa-miR-6786-5p	-	3.76×10^{-4}	Not confirmed
hsa-miR-6802-5p	-	1.11×10^{-4}	Confirmed
hsa-miR-6803-5p	-	1.76×10^{-2}	Not confirmed
hsa-miR-6805-5p	-	4.14×10^{-2}	Confirmed
hsa-miR-6816-5p	-	4.96×10^{-3}	Confirmed
hsa-miR-6869-5p	-0.033	1.45×10^{-3}	Confirmed
hsa-miR-6885-5p	-0.056	1.95×10^{-8}	Confirmed
hsa-miR-7111-5p	0.011	4.29×10^{-1}	Not confirmed
hsa-miR-762	0.0003	7.70×10^{-1}	Confirmed
hsa-miR-7704	-	2.07×10^{-2}	Confirmed
hsa-miR-7845-5p	1.698	7.15×10^{-1}	Confirmed
hsa-miR-7847-3p	-	7.09×10^{-1}	Confirmed
hsa-miR-8072	-	3.42×10^{-1}	Confirmed

Table S4 A urinary miRNA ensemble composed of 40 miRNA species for classification between 24 stage I lung cancer and 25 non-cancer subjects.

MicroRNAs	Weight coefficient	p value	EV miRNAs confirmed by ExoCarta (released in July 2015) or Vesiclepedia (released in September 2023)
hsa-let-7e-5p	-0.4916616	1.31×10^{-4}	Confirmed
hsa-mir-106a-3p	0.2198091	3.20×10^{-2}	Confirmed
hsa-mir-1250-5p	0.38910012	2.49×10^{-8}	Confirmed
hsa-mir-1265	0.02028816	8.25×10^{-6}	Not confirmed
hsa-mir-20a-3p	-3.014025	1.95×10^{-16}	Confirmed
hsa-mir-30e-3p	-0.7914333	1.22×10^{-7}	Confirmed
hsa-mir-3126-5p	0.19274675	6.59×10^{-4}	Not confirmed
hsa-mir-3164	1.06239609	1.42×10^{-7}	Confirmed
hsa-mir-339-5p	-1.6905226	1.96×10^{-7}	Confirmed
hsa-mir-378d	0.06752894	2.98×10^{-1}	Confirmed
hsa-mir-3913-3p	0.41151482	9.80×10^{-3}	Confirmed
hsa-mir-4262	0.14678614	3.83×10^{-4}	Not confirmed
hsa-mir-4307	1.12674427	2.26×10^{-9}	Not confirmed
hsa-mir-4311	1.04797204	8.68×10^{-7}	Confirmed
hsa-mir-4453	1.17142828	2.15×10^{-8}	Confirmed
hsa-mir-4491	0.45602348	2.54×10^{-4}	Not confirmed
hsa-mir-449c-5p	1.44450258	1.24×10^{-5}	Confirmed
hsa-mir-450a-2-3p	-1.417064	1.16×10^{-5}	Confirmed
hsa-mir-4520-3p	0.51308889	1.14×10^{-7}	Confirmed
hsa-mir-452-3p	-0.7470428	6.05×10^{-10}	Confirmed
hsa-mir-4529-5p	0.19114809	1.72×10^{-5}	Not confirmed
hsa-mir-4638-3p	0.65779458	3.05×10^{-3}	Confirmed
hsa-mir-6070	0.92679243	1.84×10^{-7}	Not confirmed
hsa-mir-624-3p	-0.3289615	1.69×10^{-6}	Confirmed
hsa-mir-630	-0.4452697	6.05×10^{-5}	Confirmed
hsa-mir-640	-0.4107503	3.14×10^{-7}	Not confirmed
hsa-mir-642a-5p	-0.4642681	5.73×10^{-10}	Confirmed

hsa-mir-6828-5p	1.09639229	8.76×10^{-5}	Not confirmed
hsa-mir-6837-3p	-0.4870758	3.40×10^{-9}	Confirmed
hsa-mir-938	-2.5933995	1.60×10^{-7}	Not confirmed

Table S5 Participant characteristics for brain tumor, lung cancer, and non-cancer subjects.

Characteristics	Total (N=319)
Brain tumors, N	110
Median age, years (range)	53 (2-86)
Sex, N (%)	
Male	56 (50.9)
Female	54 (49.1)
Diagnosis	
Glioblastoma, N (%)	26 (23.6)
Glioblastoma, IDH-mutant	7
Glioblastoma, IDH-wildtype	19
Lower grade glioma, N (%)	34 (30.9)
Diffuse astrocytoma, IDH-mutant	8
Diffuse astrocytoma, IDH-wildtype	6
Anaplastic astrocytoma, IDH-mutant	3
Anaplastic astrocytoma, IDH-wildtype	4
Oligodendroglioma, IDH-mutant and 1p/19q-codeleted	11
Anaplastic oligodendroglioma, IDH-mutant and 1p/19q-codeleted	2
Meningioma, N (%)	27 (24.5)
WHO grade I	26
grade II	0
grade III	1
Other brain tumors, N (%)	23 (20.9)
Lung cancer, N	93
Median age, years (range)	61 (31-80)
Sex, N	
Male	87
Female	6
Clinical stage, N	
IA	9
IB	14
IIA	10
IIB	7

IIIA	26
IIIB	10
IV	15
Unknown	2
Smoking status, N	
Never smoked	3
Former smoker	45
Current smoker	45
Non-cancer controls, N	116
Median age, years (range)	42 (20-69)
Sex, N	
Male	50
Female	66
Smoking status, N	
Never smoked	116
Former smoker	0
Current smoker	0
